# Intensive Running Enhances NF-κB Activity in the Mice Liver and the Intervention Effects of Quercetin

**DOI:** 10.3390/nu12092770

**Published:** 2020-09-11

**Authors:** Chao Gao, Yang Liu, Chunjie Jiang, Liang Liu, Juan Li, Dan Li, Xiaoping Guo, Zhu Wang, Yuexin Yang, Liegang Liu, Ping Yao, Yuhan Tang

**Affiliations:** 1 Key Laboratory of Trace Element Nutrition of National Health Commission, National Institute for Nutrition and Health, Chinese Center for Disease Control and Prevention, Beijing 100050, China; gaochao@ninh.chinacdc.cn (C.G.); liuyang@ninh.chinacdc.cn (Y.L.); wangzhu@ninh.chinacdc.cn (Z.W.); yuexin_yang@sina.com (Y.Y.); 2 Department of Nutrition and Food Hygiene, School of Public Health, Tongji Medical College, Huazhong University of Science and Technology, Wuhan 430030, China; jcj2010150044@163.com (C.J.); lijuan107@163.com (J.L.); lidangoodgirl@163.com (D.L.); momodeal@163.com (X.G.); lgliu@mails.tjmu.edu.cn (L.L.); yaoping@mails.tjmu.edu.cn (P.Y.); 3 College of Food Science and Engineering, Qingdao Agricultural University, Qingdao 266109, China; liuliangwh@163.com

**Keywords:** quercetin, intensive exercise, inflammation, liver damage

## Abstract

Background: Emerging evidence has supported that intensive exercise induces weakened performance and immune and metabolic disorders. We systematically evaluated the effects of quercetin against hepatic inflammatory damage caused by repeated intensive exercise and explored the potential mechanism. Methods: Male BALB/c mice were administered quercetin (100 mg/kg BW) for four weeks, and performed a treadmill running protocol of 28 m/min, 5° slope, 90 min/day concurrently for the last seven days. Results: Quercetin administration reduced the leakage of aspartic acid and alanine aminotransferase and improved ultrastructural abnormalities such as swelling, and degeneration caused by high-intensity running in mice. Quercetin significantly decreased the hepatic and plasmatic levels of inflammatory cytokines IL-1β, IL-6, TNF-α, inducible nitric oxide synthase, cyclooxygenase-2 and intercellular adhesion molecule-1—provoked by over-exercise. Furthermore, diminished activation and nuclear translocation of NF-κB were found after quercetin treatment through inhibiting IKKα and Iκbα phosphorylation of intensive running mice. Conclusion: Quercetin offers protection for mouse livers against intensive sports-induced inflammatory injury, and the suppression of the NF-κB signal transduction pathway may play a role in its anti-inflammatory effects. Our findings broaden our understanding of natural phytochemicals as a promising strategy to prevent excessive exercise damage.

## 1. Introduction

Regular physical exercise is generally deemed to play a protective effect on human against inflammation [1]. However, mounting evidence suggests that intensive exercise induces weakened performance, immune dysfunction and metabolic disorders [2]. The liver plays a substantial role in metabolism and possesses various functions of glycogen storage, glycogenesis and erythrocytes of decomposition, which is crucial for sports performance. On the other hand, the liver also may be susceptive to intensive exercise because of sustained energy depletion and violent metabolism disturbance [3]. Nuclear factor-κB (NF-κB)-mediated inflammatory responses have been presumed as crucial mechanisms involved in triggering liver damage following intensive exercise. It is documented that proinflammatory cytokines, reactive oxygen species, DNA damage, pathogen exposure and physical stress may activate protein kinases and then phosphorylate IκB, causing NF-κB nucleus translocation [4,5]. The activated transcription of inflammatory mediators stimulated by translocation of NF-κB causes tissues proinflammatory response [6,7]. Emerging studies have demonstrated that unaccustomed exercise induces muscle inflammatory damage, pain and performance deficits through the NF-κB pathway [8]. To date, however, hepatic inflammatory stress following exercise remains unclear—especially inflammatory damage mechanisms involving NF-κB during repeated and high-intensity exercise—which is routine for soldiers, athletes and ultra-endurance sportspeople.

As one of the most common flavonoids, quercetin (3,3’,4’,5,7-pentahydroxyflavone), has been collectively elucidated to exhibit many beneficial effects, such as anti-inflammatory, immunoregulatory, antipathogenic and antioxidative activity [9,10]. Some research suggests that quercetin promotes mitochondrial biogenesis and increases VO_2max_-endurance exercise performance in vivo models [11,12,13], despite some adverse reports [14,15]. Our previous study observed a definite antioxidative role of quercetin in intensive running-caused malfunctions in mice myocardial mitochondria [16]. Moreover, quercetin can exert an anti-inflammatory role similar to some conventional nonsteroidal anti-inflammatory drugs (NSAIDs) without any apparent side effects, for example, cardiovascular complications and gastrointestinal distress [17]. Anti-inflammatory mechanisms of quercetin may be linked to its inhibiting effects on the inflammatory cascade, particularly the NF-κB signaling pathway [18,19]. However, there has been little attention paid to the role of quercetin in liver damage caused by repeated strenuous exercise—especially inflammatory injury and hepatic NF-κB signal transduction pathway. This study aimed to investigate the beneficial role and the underlying molecular mechanism of quercetin on intensive running-induced liver damage, with the NF-κB pathway as the main focus.

## 2. Materials and Methods

### 2.1. Chemicals and Materials

Quercetin (≥98%, HPLC) was obtained from Sigma-Aldrich. Mouse β-actin antibody and horseradish peroxidase (HRP) conjugated goat anti-mouse IgG were obtained from Sigma-Aldrich (St. Louis, MO, USA) and Cell Signal (Danvers, MA, USA), respectively. Mouse iNOS, COX2 and ICAM-1 antibodies were obtained from Santa Cruz (CA, USA). Assay kits for aspartate/alanine transaminase (AST/ALT) were purchased from Mindray (Shenzhen, China). TNF-α, IL-1β, IL-6 and IL-10 ELISA kits were provided by Joyee Biotechnics (Shanghai, China). Assay kits for nuclear and cytoplasmic protein extraction and LightShift chemiluminescent EMSA were obtained from Beyotime Institute of Biotechnology (Haimen, China) and Pierce Biotechnology (Waltham, MA, USA), respectively. Local reagent retailers provided additional analytical grade chemicals.

### 2.2. Animals Treatment and Exercise Protocol

BALB/C mice (8 weeks old, 18 ± 1 g, male) were acclimated for one week and then randomly divided into four groups (eight mice each group): rested controls (Ct), intensive exercise (Ex), intensive exercise + quercetin administration (Ex + Qu) and rested + quercetin administration (Qu). Quercetin was supplemented for 4 weeks by gavage at a dose of 100 mg/kg·bw every day, and an intensive running protocol was performed at the fourth week [20,21]. The mice were acclimated to running on a motor-driven treadmill for two successive days, beginning at 10 m/min on a 5° slope for 10 min/day and then submitted to daily sessions of intense exercise for seven consecutive days (the fourth week) following a 2-day rest. The daily sessions of intensive training set of 28 m/min at a 5° slope for 90 min following a 10 min warm-up. The mice were encouraged to exercise with a soft brush when they were exhausted [21]. We attached the flow chart of the experiment design to the Supplementary Materials (Figure S1). The experiment was subject to approval by the Tongji Medical College Council on Animal Care Committee (IACUC number: S407). Tap water and rodent laboratory chow were available ad libitum. Blood was sampled from the mice’s eyes; the serum was obtained from the blood by centrifuging at 3500 g for 10 min at 4 °C. Liver samples were collected quickly to fix for morphologic examination (the central zone of the medial lobe) or to freeze at −80 °C for further assays.

### 2.3. Liver Pathomorphological Examination

Fresh liver sample slices were fixed with paraformaldehyde and then dehydrated and embedded in paraffin. Approximately 4-μm liver tissue sections were stained with hematoxylin and eosin and observed with a light microscope. Meanwhile, another smaller fresh liver fragment (1-mm cube) was fixed in turn with 2.5% glutaraldehyde and 1% osmium tetroxide in a pH 7.4 phosphate buffer. After dehydration, the sample was embedded in resin and ultrathin sections were stained with uranyl acetate and lead citrate for ultrastructure examination by a transmission electron microscope.

### 2.4. Serum Assays by Biochemistry and ELISA

AST and ALT were analyzed by enzymatic kinetic method. Serum TNF-α, IL-1β, IL-6 and IL-10 levels were measured using enzyme-linked immunosorbent assay (ELISA) kits. Absorbance was measured spectrophotometrically with a SpectraMax M2 microplate reader; the concentration of each cytokine was calculated by comparison with a calibration curve.

### 2.5. Liver RNA Extraction and qRT-PCR Analysis

The target mRNAs were quantified by a 7900HT real-time PCR system with SYBR green-based qRT-PCR kit and specific oligo primers. The β-actin mRNA level was quantified as an endogenous control. The relative mRNA expression was expressed as fold-change relative to rest control. The forward and reverse primers were as follows: TNF-α (NM_013693): CATCTTCTCAAAATTCGAGTGACAA Moreover, TGGGAGTAGACAAGGTACAACCC; IL-1β (NM_008361.3): CTTCAGGCAGGCAGTATCACTC and TTGTTGTTCATCTCGGAGCC; IL-6 (NM_031168.1): CCACGGCCTTCCCTACTTC and CTCATTTCCACGATTTCCCAG; IL-10 (NM_010548): GGTTGCCAAGCCTTATCGGA and ACCTGCTCCACTGCCTTGC; β-actin (NM_007393.3): CTGAGAGGGAAATCGTGCGT and CCACAGGATTCCATACCCAAGA.

### 2.6. Protein Detection by Immunohistochemistry and Western Blot

Liver tissue sections (4 μm) embedded with paraffin from the pathomorphological examination were incubated overnight with polyclonal rabbit anti-mouse iNOS, COX2 or ICAM-1 antibody (diluted in 1:100 at 4 °C), respectively. Following incubation with goat anti-rabbit horseradish peroxidase-conjugated secondary antibody (diluted to 1:200 for 1 h) and immunostaining with 3, 3’-diaminobenzidine, the expression of hepatic iNOS, COX-2 or ICAM-1 was observed by light microscope using integral optical density analytic method.

Liver tissues were homogenized and lysed in RIPA lysis buffer (1% deoxycholate, 1% Triton X-100, 0.1% SDS). Tissue lysates with equal amounts of about 50 μg of protein were subjected to western blotting. The target protein was probed with specific primary antibody of p-IKKα, IκBα or p-IκBα and a species-specific secondary antibody. Immunoreactive bands were analyzed with an ECL plus western blotting detection system. Quantitative analysis of band relative density was used by Gel Pro 3.0 software with correction by background and standardization to β-actin.

### 2.7. NF-κB Activation Assay by IFL, LSCM and EMSA

The immunoreactivity of NF-κB and IκBα in the liver was observed by the double immunofluorescence labeling (IFL) method. Briefly, deparaffinized and dehydrated sections (4 μm) were incubated with mouse monoclonal anti-NF-κB (Santa Cruz, CA, USA) antibody and anti-Iκbα (Epitomics, Burlingame, CA, USA) antibody overnight at 4 °C after blocking with 5% BSA and then with Alexa 594-labeled goat antibody against mouse IgG or Alexa 488-labeled goat antibody against rabbit IgG (1:400, KPL). Nuclei were stained by incubating with DAPI for 5 min. The stained sections were examined using a UltraVIEW Vox (PerkinElmer, Inc., Waltham, MA, USA) with Volocity software 5.3 for image capturing. Meanwhile, nuclear extracts were prepared from frozen liver tissues using a nuclear and cytoplasmic protein extraction kit. Meanwhile, nuclear extracts were prepared from frozen liver tissues using a nuclear and cytoplasmic protein extraction kit (Beyotime, Jiangsu, China). Activation of NF-κB was analyzed by electrophoretic mobility shift assay (EMSA) using consensus oligonucleotides of NF-κB (5’-AGT TGA GGG GAC TTT CCC AGG C-3’) as a probe. By using Biotin 3’ End DNA Labeling Kit as per the manufacturer instructions, single-stranded DNA primers was tagging with biotin (Pierce Biotechnology Waltham, MA, USA). Nuclear protein extract and biotin-labeled oligonucleotides were mixed and incubated for 20 min at room temperature. The biotin-labeled oligonucleotide-protein complex was run in 0.5 × TBE buffer non-denaturing polyacrylamide gels at 380 mA for 30 min, then transferred to a nylon membrane. After membrane cross-linking for 20 min on a UV transilluminator, biotin-labeled DNA was detected with LightShift chemiluminescent EMSA kit, and optical density of the specific bands was quantified with an imaging densitometer.

### 2.8. Statistical Analysis

Data are presented as mean ± SD. One-way analysis of variance (ANOVA, San Francisco, CA, USA) followed by Student–Newman–Keuls multiple range test was used to analyze the data with SPSS 20.0 software package (Microsoft Windows, Redmond, WA, USA). The value of statistical significance was *p* < 0.05.

## 3. Results

### 3.1. Quercetin Treatment Alleviated Liver Damage of Mice Exposed with Intensive Exercise

**Figure 1:**
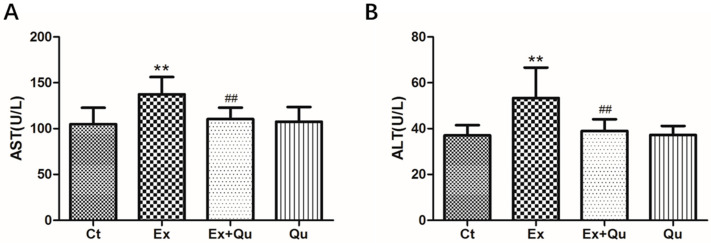
Effect of intensive running with or without quercetin administration on serum aspartate transaminase (AST) (**A**) and alanine transaminase (ALT) levels (**B**). BALB/c mice were pretreated or not with quercetin for 4 weeks and subsequently exposed to intensive exercise for successive 7 days. Results presented as mean ± SD (*n* = 8). Ct—rested control; Ex—intensive exercise; Ex + Qu—Ex and quercetin; Qu—rested + quercetin. ** *p* < 0.01 vs. Ct; ## *p* < 0.01 vs. Ex.

Food intake or weight gain was not significantly influenced by intensive exercise or quercetin treatment (data not shown). Intensive running induced a substantial increase of the mice serum ALT and AST (*p* < 0.01), suggesting obvious liver injury was driven by over-exercise. Quercetin interventions in exercising mice significantly decreased both serum ALT and AST (*p* < 0.01). Meanwhile, the effects of serumALT and AST levels by quercetin itself were not observed (Figure 1).

### 3.2. Quercetin Improved Intensive Exercise-Derived Hepatic Pathomorphological Abnormity

**Figure 2:**
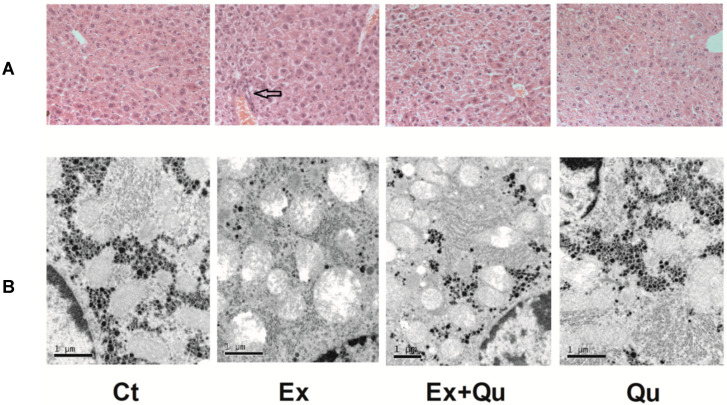
Effect of quercetin intervention on liver morphology disturbed by intensive exercise. Liver samples processed by hematoxylin–eosin were observed by Olympus BX50 light microscope with HMIAS-2000 medical imaging system (×200) (**A**). The ultrastructure of the liver was shown in a transmission electron microscope (TEM) images (**B**). Ct—rested control; Ex—intensive exercise; Ex + Qu—Ex and quercetin; Qu—rested + quercetin.

As shown in Figure 2A, hematoxylin–eosin staining of intensive exercise mice showed sustained infiltration of inflammatory cells into liver lobules, tissue destruction and erythrocyte in influx (hemorrhage), which was not observed in both rested controls. Administration of quercetin markedly attenuated histological abnormalities resulted from exhaustive exercise. The protective role of quercetin was further observed by TEM. In Figure 2B, visualized by ultrastructural examination, rested control mice were rich in normal organelles, including well-developed mitochondria with intact membrane and homogeneously distributed cristae, ribosomal localized on the tidy rough endoplasmic reticulum and uniform glycogenosomes. The hepatocytes of intensively running mice, however, exhibited evident swollen and degenerative changes. Intensive running caused a decrease in mitochondria quantity and structural heterogeneity, including destruction of mitochondrial membranes, cristae and electron–lucent matrix, together with severe swelling. RER was considerably swollen, degenerated and decreased. Glycogenosomes were significantly reduced and even vanished. Intensive exercise-induced abnormal ultrastructural or disordered RER, and glycogenosomesmitochondria distribution was partly normalized by quercetin. However, the quantity of RER and glycogenosomes remained unchanged. Meanwhile—compared to normal control—the addition of quercetin alone had no apparent effect on hepatic ultrastructure.

### 3.3. Quercetin Alleviated Intensive Exercise-Derived Inflammatory Stress

**Figure 3:**
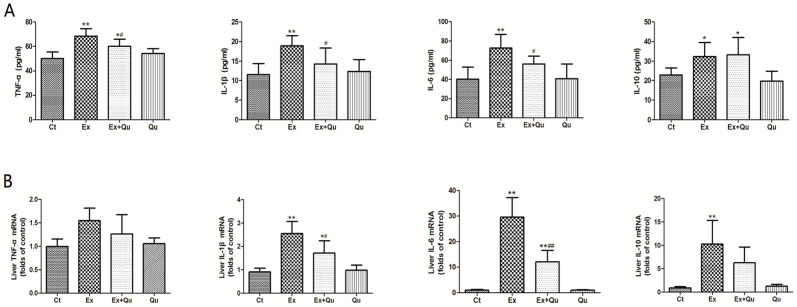
Serum and hepatic inflammatory cytokines levels of intensive exercise and/or quercetin prophylaxis. (**A**) Serum inflammatory cytokines were quantified by ELISA; results represented as mean ± SD (*n* = 8); (**B**) liver TNF-α, IL-1β, IL-6 and IL-10 levels evaluated by qRT-PCR; values presented as multiples of control following normalization by β-actin and mean ± SD (*n* = 8). Ct—rested control; Ex—intensive exercise; Ex + Qu—Ex + quercetin; Qu—rested + quercetin. * *p* < 0.05, ** *p* < 0.01 vs. Ct; # *p* < 0.05, ## *p* < 0.01 vs. Ex.

Data for serum cytokines are indicated in Figure 3. Intensive exercise remarkably increased serum levels of TNF-α, IL-1β and IL-6—and quercetin pretreatment improved the adverse effects. Notably, increased anti-inflammatory factor IL-10 was discovered in the exercise group, but no significant improvement occurred on IL-10 level after quercetin treatment. Quercetin itself had no impact on the level of serum cytokines compared to intact normal control. Likewise, repeat intensive exercise resulted in increased hepatic mRNA levels of TNF-α, IL-1β, IL-6 and IL-10. Quercetin pretreatment showed significant effect on IL-1βand IL-6 and insignificant effect on TNF-α and IL-10mRNA levels enhanced by intense exercise.

**Figure 4:**
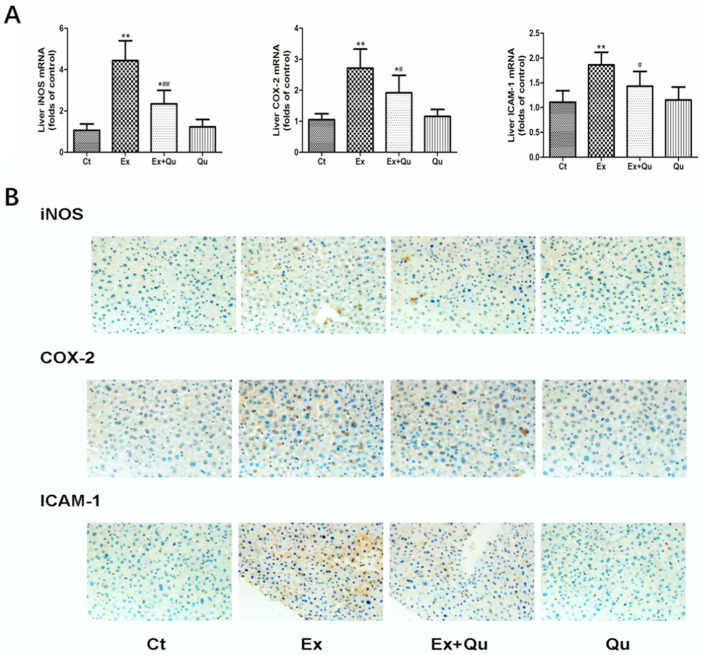
Quercetin decreased the expression of iNOS, COX-2 and ICAM-1 in mice liver subjected to intensive exercise. (**A**) mRNA expression determined by real-time PCR following normalization to β-actin; data expressed as fold-change compared to the control group (mean ± SD, n = 8); (**B**) protein expression determined by immunohistochemistry and observed by Olympus BX50 light microscope with HMIAS-2000 medical imaging system (×200). Ct—rested control; Ex—intensive exercise; Ex + Qu—Ex + quercetin; Qu—rested + quercetin. * *p* < 0.05, ** *p* < 0.01 vs. Ct; # *p* < 0.05, ## *p* < 0.01 vs. Ex.

Further, exercise resulted in a notable rise of hepatic iNOS, COX-2 and ICAM-1 mRNA levels by 3.2-fold, 1.6-fold and 0.7-fold, respectively, whereas these negative changes were partially blinded by quercetin administration (Figure 4A). Moreover, hepatic protein levels of iNOS, COX-2 and ICAM-1 showed similar influence as the findings on mRNA levels following over-exercise or quercetin intervention (Figure 4B).

### 3.4. Quercetin Ameliorated Exercise-Induced Inflammatory Stress through Suppressing NF-κB Activation

**Figure 5:**
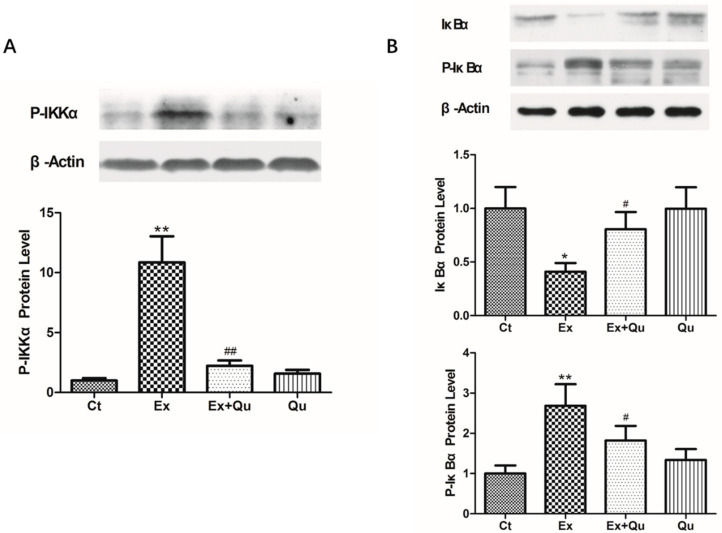
Phosphorylated protein level of IKKα (**A**), IκBα and P-IκBα (**B**) in mice liver exposed to intensive running with or without quercetin pretreatment. Figures show representative western blot (upper panel) and densitometric analysis (lower panel). Data presented as fold-change compared to the control group. Results represent mean ± SD (*n* = 8). Ct—rested control; Ex—intensive exercise; Ex + Qu—Ex + quercetin; Qu—rested + quercetin. * *p* < 0.05, ** *p* < 0.01 vs. Ct; # *p* < 0.05, ## *p* < 0.01 vs. Ex.

Given the finding that nuclear translocation and activation of NF-κB often coincides with increased protein degradation of IkBα regulated primarily by IKKα [22], we measured the protein expression of IkBα (unphosphorylated and phosphorylated forms) and its crucial regulating kinase IKKα. As is illustrated in Figure 5, exhausting exercise generated a decline of the nonphosphorylated way (–0.59-fold), while phosphorylated IκBα protein level was significantly raised (+1.7-fold). Accordingly, IKKα protein was upregulated in exercised mice by 9.9-fold, compared with the rested control (*p* < 0.01). IκBα phosphorylation and IKKα upregulation induced by repeated intensive exercise was partially abolished by quercetin prevention. However, quercetin itself did not affect IκBα phosphorylation and IKKα expression in contrast to the rested control.

**Figure 6:**
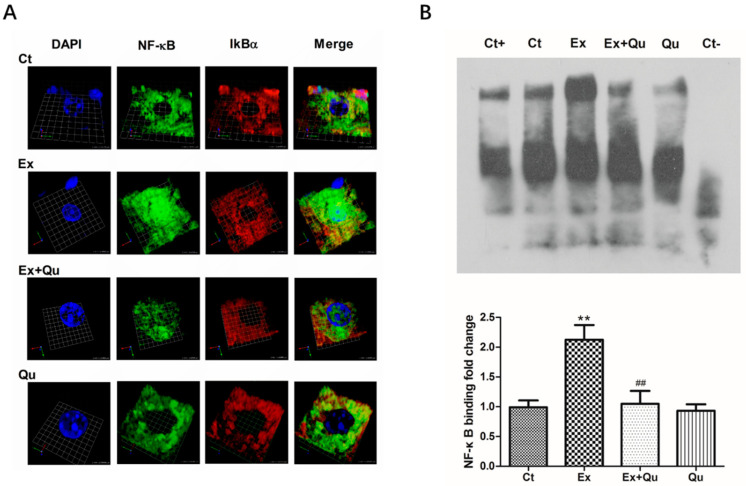
Effect of quercetin on the nuclear expression level and activity of NF-κB in mice liver following intensive running. (**A**) Liver sections were probed with NF-κB (green) and Iκbα (red) antibodies, while DAPI was used to stain the nuclei (blue); (**B**) activity of NF-κB-DNA binding was quantified by densitometry. Specific binding was verified by the addition of unlabeled (cold) oligonucleotide (competitor, Ct-) or labeled oligonucleotide mutate (noncompetitor, Ct+). Representative EMSA (upper panel) and densitometric analysis (lower panel). Data presented as fold-change in contrast to the control group. Values presented as mean ± SD (*n* = 8). Ct—rested control; Ex—intensive exercise; Ex + Qu—Ex + quercetin; Qu—rested + quercetin. ** *p* < 0.01 vs. Ct; ## *p* < 0.01 vs. Ex.

NF-κB translocation was also assessed by immunofluorescence using a laser scanning confocal microscope (LSCM). Note that in the rested control and quercetin controls, NF-κB was not found concentrated in the hepatocyte nuclei (stained with DAPI, left). Also note that in the exercised mouse that the colocalization of NF-κB (green) with several nuclei (blue). As shown in Figure 6A, we found that more green fluorescence (NF-κB) was colocated with blue-fluorescent nuclei in over-exercised mice compared with rested control, indicating an evident translocation of activated NF-κB into nuclei. Such translocation was suppressed by quercetin pretreatment to exercise-challenged mice. To analyze the nuclear translocation of NF-κB more clearly, we used Volocity software 5.3 (Quorum Technologies, Ltd., Lewes, UK) software to create a dynamic video rendering of the three-dimensional spatial structure of a single mouse hepatocyte and attached it to the Supplementary Materials.

As shown in Figure 6B, by EMSA assay, the DNA-binding activity of NF-κB was almost entirely abolished by unlabeled oligonucleotide competitor (Ct-) and partially lowered by nonspecific oligonucleotide mutate (Ct+), indicating a specific binding between designed probe and NF-κB. The capacity of DNA-binding of NF-κB induced by repeated exhausted-exercise increased 1.15-fold relative to rested control (*p* < 0.01). Quercetin alone did not affect the basic DNA-binding capacity of NF-κB of rested control, but nearly wholly neutralized exercise-induced DNA-binding to the normal level when pretreated to exercised mice by quercetin. The NF-κB binding activity was investigated by EMSA performed with an NF-κB consensus nucleotide sequence.

## 4. Discussion

The liver plays a central role in metabolism and has a series of essential functions crucial for sports performance and recovery after over-training. In this study, continuous intensive running resulted in a substantial release of hepatic aminotransferases, inflammatory cell infiltration, tissue destruction and congestion. Morphological changes shown by TEM indicated subcellular damage with extensively swollen, cristae-disrupted, membrane-fragmented mitochondria, degenerated RER and decreased glycogenosomes. Correspondingly, we found that over-exercise dramatically increased expression of hepatic mRNA and serum proinflammatory cytokines such as TNF-α, IL-1β and IL-6 and initiated hepatic inflammatory cascades involving iNOS, COX-2 and ICAM-1. These findings collectively showed that intensive exercise resulted in sustained inflammatory damage in mice livers, which is partially in line with other observations [23]. Cheney [24] et al. found that strenuous exercise can lead to liver injury and that *Clostridiales* in mice and extravascular hemolysis by free iron may involve in liver damage. In this paper, quercetin effectively ameliorated this exercise-derived inflammatory damage. Our previous study has shown that quercetin protects the mouse liver from inflammatory stress caused by toxic insults [25].

Inflammatory reactions, their initiation and aggravation of each other may be thought of as one of the most crucial mechanisms to trigger tissue damage following overtraining or overstrain. Our data showed that continuous exhausted exercise caused significant inflammatory stress and pathological malformation in the mouse liver. As a pleiotropic transcription factor, NF-κB has been increasingly regarded as a key player in exercise-caused inflammation [26]. There are five structurally related subunits in the Rel/NF-κB family of eukaryotic transcription factors. RelA (p65), cREL and RelB contain transactivation domains with the capacity of initiating gene transcription, while p50/p105 and p52/p100 generally function in the inactivation of the gene for the lack of a transactivation domain. The p65/p50 heterodimer is considered as a decisive classical member of the NF-κB family with two subunits, its p65 subunit executing transcription-initiating function and another inducing DNA-binding only [27]. Inhibited by binding to IκBs, NF-κB remains inactivated and stay within the cytoplasm under normal physiological status. Upon stimulation—such as in intensive exercise—IκBs is phosphorylated by activated IKK, followed by the 26 S proteasome complex-mediated polyubiquitination and degradation. Freed from its sequestrator, NF-κB enters the nucleus and transactivates NF-κB-responsive genes [28,29]. Ji [22] et al. provided evidence that exhaustive exercise upregulates NF-κB expression in muscles and cytosolic IKK and IκBα content was concomitantly decreased. Vella [30] et al. reported that resistance exercise resulted in decreased IκBα protein expression in the cytoplasm, which was coincident with the increase and nuclear translocation of phospho-NF-κB in human skeletal muscle. Our data extended the findings from skeletal muscle and demonstrated the activation of NF-κB as a result of intensive running supported by increased nuclear translocation and DNA-binding activity in the liver.

Activation of NF-κB involves a series of genes dysregulation in the expression of the inflammatory factors, some of which have become potential targets for prophylaxis and treatment. Previous studies have shown the crucial roles of NF-κB activation plays in iNOS expression in gastrocnemius muscle [29] and skeletal muscle cells [31] in rats. It is still undetermined whether the expression of COX-2 and ICAM-1 has a relation with NF-κB activation caused by exercise in the liver. However, it has been evidenced that NF-κB regulates the expression of adhesion molecules during leukocyte migration [32] and that the ICAM-1 promoter could be bind to the NF-κB binding site [33]. As expected, our research revealed that quercetin attenuated inflammatory stress and pathological malformation in mouse liver caused by continuous exhausting exercise, possibly by mainly suppressing nuclear translocation of NF-κB and the release of proinflammatory mediators. The regulation of cellular signaling molecules, such as NF-κB, AP-1 and COX-2 and transcription factors involved in inflammation inhibiting mechanism of quercetin. The direct inhibitory effect of quercetin on oxidative stress and NF-κB activation has been observed in gastric mucosa of portal hypertensive rats [34]. Moreover, quercetin inhibits NF-κB translocation by directly inhibiting IKK-mediated phosphorylation and degradation of IκB, which in turn results in a decreased transcription of various inflammatory cytokines, participating in its underlying anti-inflammatory effects [35,36,37]. Inhibition of AP-1 activation by quercetin is thought to be achieved by competitively binding to its DNA motif or inhibiting the components (*c-FOS* and *C-Jun*) in the AP-1 pathway [16]. In addition, ROS-scavenging capacity of quercetin may as well contribute to its inactivation of NF-κB and AP-1 [9,10]. Garcia-Mediavilla [35] et al. illustrated downregulated expression of COX-2/iNOS and NF-κB pathway after quercetin treatments in Chang liver cells. Although the mechanism by which quercetin induces COX-2 reduction has not been elucidated, NF-κB and AP-1 are considered to exact essential functions [16]. Unlike NSAID drugs, which specifically inhibit COX-2, it seems quercetin’s effects are more targeted explicitly at NF-κB with less potential side effects [38,39]. Nevertheless, although the data provide evidence indicating that quercetin may serve anti-inflammation functions through inhibition of NF-κB, it also seems that the direct scavenging and inactivation of superoxide, hydroxyl radical or peroxynitrite or poly (ADP-ribose) activation ability of quercetin may also have beneficial effects on blocking inflammatory process. It has been shown that ROS is not only involved in oxidative stress, but also has a role in inflammation process by activating transcription factors such as NF-κB and AP-1 to induce the production of cytokines of TNF-α and IL-6 [40].

## 5. Conclusions

Whether physical exercise is harmful—or can even lead to long-term damage to liver function—needs to be taken seriously. Therefore, more studies on the causes and effects of exercise-induced liver stress and the biologic effects of inflammatory mediators are urgently needed. Our research provides novel evidence that quercetin has an ambiguous anti-inflammatory protective effect on liver injury and dysfunction following repeated acute exercise by inhibiting NF-κB activation and subsequently proinflammatory mediator release. The protective effect of quercetin pretreatment provides a promising strategy of functional food development, especially naturally occurring phytochemicals for high-intensity exercise-induced liver injury, while the exact pharmacological mechanism remained to be fully clarified.

## Supplementary Materials

The following are available online at https://www.mdpi.com/2072-6643/12/9/2770/s1, Figure S1: Flow chart of the experiment design, Video S1: Ct—rested control: three-dimensional spatial structure of NF-κB nuclear translocation in a single mouse hepatocyte, Video S2: Ex—intensive exercise: three-dimensional spatial structure of NF-κB nuclear translocation in a single mouse hepatocyte, Video S3: Ex + Qu—Ex + quercetin: three-dimensional spatial structure of NF-κB nuclear translocation in a single mouse hepatocyte.
