# Supplementary figures and images for: Intensive Running Enhances NF-κB Activity in the Mice Liver and the Intervention Effects of Quercetin
Source: Nutrients. 2020 Sep 11;12(9):2770. doi: 10.3390/nu12092770 (PMC7551556; doi:10.3390/nu12092770)

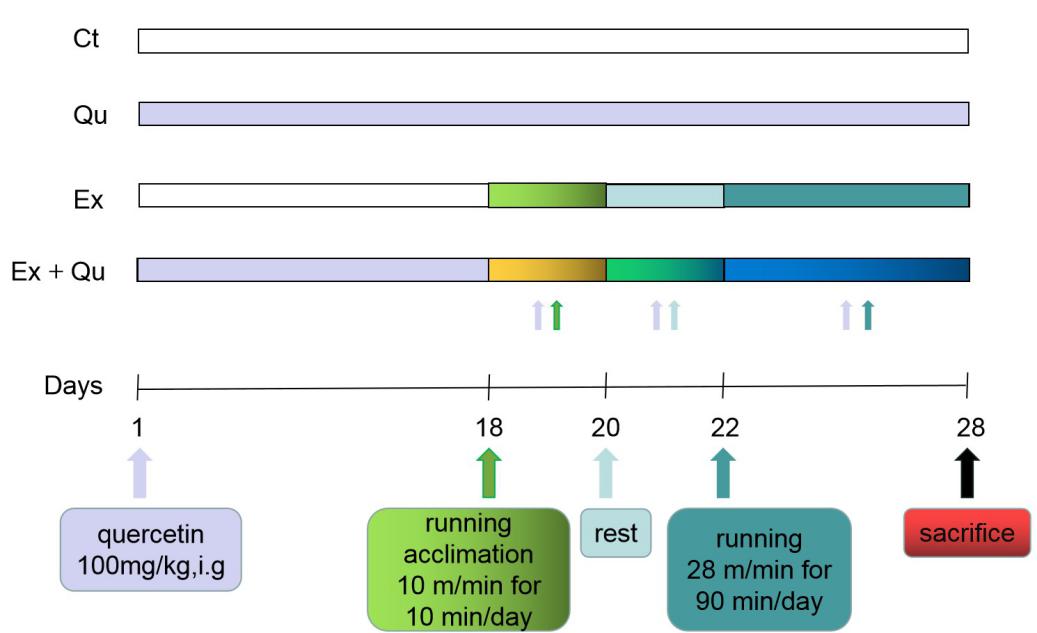


**Figure S1.** Flow chart of the experiment design.

Supplement: Supplementary file 1 [file nutrients-12-02770-s001.zip › nutrients-849913-supplementary/Figure S1.docx]
